# Mantle cell lymphoma: A clinical review of the changing treatment paradigms with the advent of novel therapies, and an insight into Indian data

**DOI:** 10.1002/cnr2.1590

**Published:** 2021-11-24

**Authors:** Vivek Sulekha Radhakrishnan, Padmaja Lokireddy, Mayur Parihar, Prashanth Srirangapattana Prakash, Hari Menon

**Affiliations:** ^1^ Clinical Hematology Oncology and HCT Tata Medical Center Kolkata India; ^2^ Hemato‐Oncology and Stem Cell Transplant Apollo Hospitals Hyderabad India; ^3^ Laboratory Hematology and Cytogenetics Tata Medical Center Kolkata India; ^4^ Medical Affairs AstraZeneca Pharma India Limited Bangalore India; ^5^ Hemato‐Oncology and Bone Marrow Transplant Cytecare Hospitals Bangalore India

**Keywords:** acalabrutinib, BTK inhibitors, guidelines for mantle cell lymphoma, ibrutinib, India, mantle cell lymphoma, novel therapies, small molecule inhibitors, targeted therapies

## Abstract

**Background:**

Mantle cell lymphoma (MCL) is a rare type of mature B‐cell lymphoid malignancy with the pathologic hallmark of translocation t(11;14) (q13, q32), which leads to an overexpression of Cyclin D1 (CCND1). The disease is also characterized by the presence of a high number of recurrent genetic alterations, which include aberrations in several cellular pathways. MCL is a heterogeneous disease with a wide range of clinical presentations and a majority presenting with aggressive disease in advanced stages.

**Recent findings:**

Management of MCL is bereft with challenges due to its resistant and relapsing pattern. Despite improvements in remission durations, the disease is currently incurable with standard therapy and has a median survival of about 3–5 years. The use of small molecules like the bruton tyrosine kinase (BTK) and BCL2 inhibitors, for treating relapsed MCL has been established leading to a diminishing role for conventional chemotherapy. Combinations of small molecule inhibitors with or without chemoimmunotherapy, are showing promising results. Cellular therapy in the form of CAR‐T cell therapy, has been approved recently.

**Conclusions:**

Personalized cancer treatment and chemo‐free regimens are showing promise and results from well‐planned long‐term studies are evolving. In India, there is a paucity of epidemiological, clinical, and research data in this field.

## 1 INTRODUCTION

Mantle cell lymphoma (MCL) is a mature B‐cell neoplasm and accounts for 5% of all non‐Hodgkin's lymphomas (NHL).^1^ It is morphologically, phenotypically, and genetically well defined. The annual incidence of MCL is around 1 in 200 000 with a male predominance (3:1). The median age of diagnosis is around 71 years.^2^ In India, 6% of all newly diagnosed NHL are MCL with a median age of diagnosis around 57 years.^3^ The two subtypes of MCL as defined by the 2016 revision of the WHO Classification of lymphoid neoplasms, the Indolent and Classic variants, have variable clinical course and require varied approaches for their management. The decision on management is also influenced by other factors like the age of the patient, functional status, comorbidities, morphology, disease stage, cost and access to drugs, tolerance to treatment and availability of clinical trials for patients among others.^4^

This article attempts to review the presentation and management of MCL, with a glimpse into its pathophysiology, and emphasis on treatment options with the current role and impact of novel therapies. We also reflect on the data available from Indian centres.

## 2 PATHOPHYSIOLOGY

**FIGURE 1:**
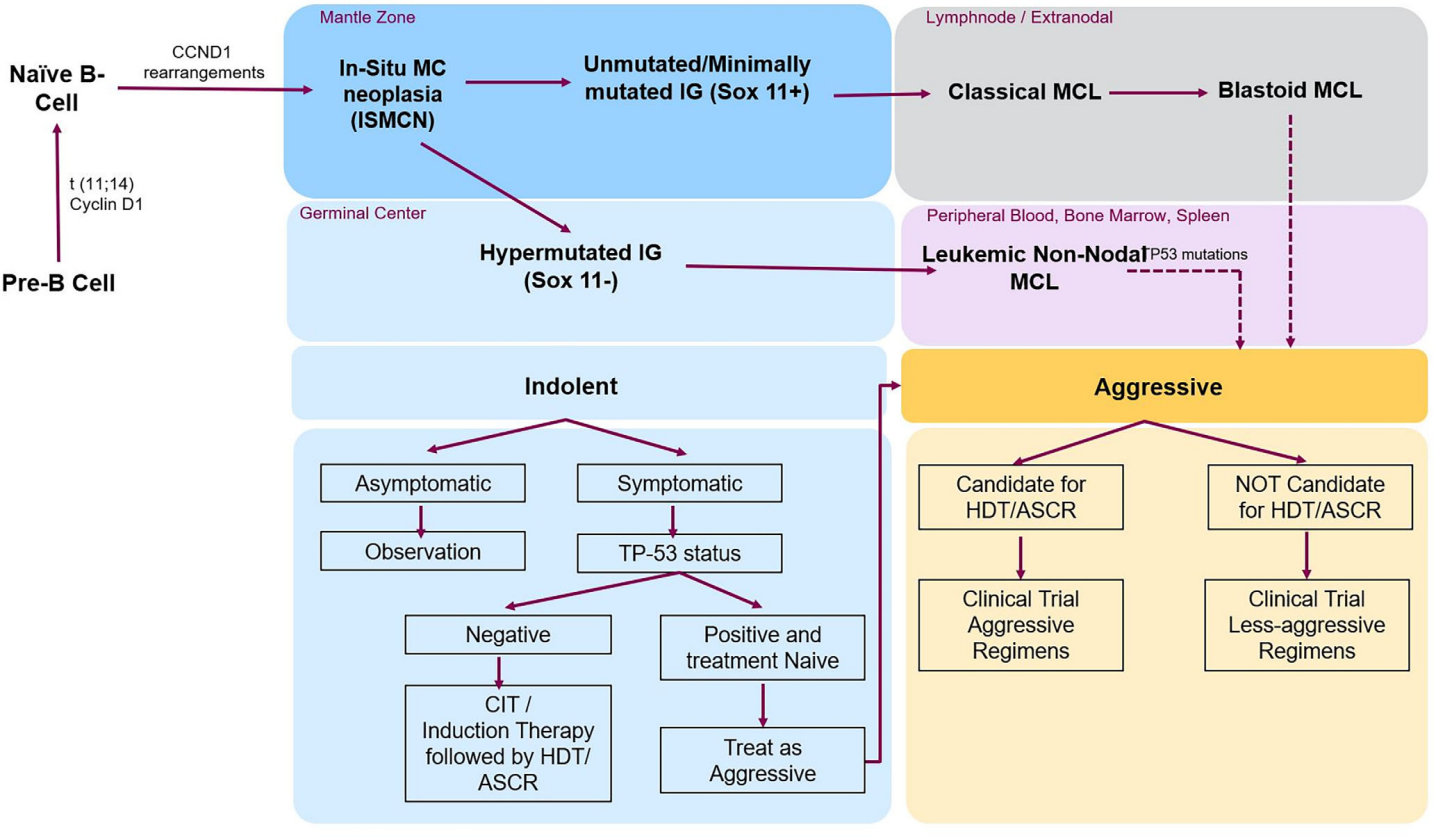
Schematic diagram of pathophysiology of MCL and outline of management options. ASCR, autologous stem cell rescue; CIT, chemoimmunotherapy; HDT, high dose therapy; IG, immunoglobulin; ISMCN, in‐situ mantle cell neoplasia; MC, mantle cell; MCL, mantle cell lymphoma

MCL is a lymphoproliferative disorder characterized by proliferation of mature B‐lymphocytes, with most cases derived from naïve pre‐germinal center and some, developing from post‐germinal Centre B‐cell populations. The pathologic hallmark of MCL is the presence of t (11;14) (q13, q32) translocation leading to an overexpression of Cyclin D1 (CCND1) which deregulates the cell cycle progression at G1‐S checkpoint by overcoming the suppressor effect of retinoblastoma 1 (RB1) and the cell cycle inhibitor p27.^5^ In less than 5% cases, a variant CCND1 translocation with immunoglobulin (IG) light chains kappa and lambda, leading to over expression of Cyclin D1 has been noted.^6^ The 2016 revision of the WHO classification of lymphoid neoplasms identifies two different pathways for development of MCL.^7^ CCND1 overexpression by itself is incapable of driving the malignant process in MCL. A myriad of secondary genetic and epigenetic changes leads to alterations in the key signaling pathways, which contribute to malignant transformation. Figure 1 shows a schematic diagram of the pathogenesis of MCL with an outline of the management options based on disease state.

In situ *Mantle‐cell Neoplasia*: This is a rare lesion of uncertain clinical significance and most often ‘incidentally’ diagnosed. It is characterized by CD5 and Cyclin D1 positive cells in the mantle zone of the lymphoid follicle in a morphologically reactive lymph node. It has an indolent course and rarely progresses to overt mantle cell lymphoma.^7^, ^8^

## 3 CLINICAL PRESENTATION

MCL has a wide range of clinical presentations. Nearly 70%–80% of MCL patients present with an aggressive disease manifesting as symptomatic lymphadenopathy or extra‐nodal disease, in an advanced stage (Stage III–IV). However, patients may also present with an indolent disease consisting of asymptomatic lymphocytosis or non‐bulky nodal/extra nodal disease, with minimal symptoms. B symptoms (unintentional weight loss, drenching night sweats, and fever) are seen in about 40% of the patients. Patients can present with abdominal distension due to hepatosplenomegaly. Lymph nodes and spleen are common sites of involvement followed by Waldeyer's ring, bone marrow and peripheral blood. Patients can also present with symptoms due to extra‐nodal involvement of GI tract, lungs, and/or CNS and orbit. An uncommon, but distinctive, presentation is the occurrence of multiple lymphomatous polyposis.^9^, ^10^

## 4 DIAGNOSIS

A complete history and physical examination undertaken to assess B symptoms, co‐morbidities and performance status of MCL patients is essential. Laboratory investigations should include a complete blood count with differential counts to assess for cytopenias secondary to bone marrow involvement, lactate dehydrogenase levels (correlates with tumor burden), complete metabolic panel, and viral serology (hepatitis and HIV panels).

Morphologically, MCL presents as a spectrum of findings, with classical tumors showing proliferation of small–medium sized lymphocytes with irregular nuclei and inconspicuous nucleoli on one end, and blastoid MCL showing cells with blastoid morphology having rounded nuclei, finely dispersed chromatin, and inconspicuous nucleoli, at the other end. Some tumors have larger cells with irregular and pleomorphic nuclei and distinct small nuclei. The Blastoid and Pleomorphic variants of MCL have an aggressive histology with high proliferation rates and the transformed variants have a high degree of aneuploidy and exhibit KMT2B, KMT2D and other mutations. These variants have an aggressive clinical behavior.^11^ Rare variants include small cell and marginal zone like MCL.

Immunophenotyping of tumor cells by multi‐parameter flow cytometry or immunohistochemistry tumor tissue biopsy, bone marrow sample or peripheral blood helps differentiate MCL from other B cell tumors and confirms diagnosis. Lymph node biopsy is preferred over aspiration. A typical immunophenotyping report by flow cytometry and immunohistochemistry report on tissue biopsy of MCL will be positive for CD5 (a T‐cell‐associated antigen), CD20, CD19, FMC7, sIgM/sIgD, CD22, CD79b and strongly Cyclin D1, and negative for CD23 (a key cell surface molecule for B‐cell activation and growth) and CD10 (a germinal centre‐associated antigen). MCL phenotype can be confused with a CLL immunophenotype. Table 1 shows the differences between MCL and different Lymphomas.^12^, ^13^

**TABLE 1:** Immunophenotypic markers in different B‐cell malignancies

Histologic subtype	CD5	CD23	CD43	CD10	BCL6	Cyclin D1	sIg (type)	CD 20	CD200
MCL	+	−	+	−	−	+	+ (M ± D)	+	−
FL	−	−/+	−	+/−	+	−	+ (G ± M)	+	+/−
SLL/CLL	+	+	+	−	−	−	+ (M ± D)	+	+
LPL	−	−	−/+	−	−	−	+/− (M)	+	
SMZL	−	−	−	−	−	−	+ (M ± D)	+	+/−
EMZL (MALT type)	−	−/+	−/+	−	−	−	+ (M)	+	+/−
HCL	−	−/+		−/+	−	−	+	+	+/−

*Note*: Immunophenotypic markers in different B‐cell malignancies.

Abbreviations: EMZL, extranodal subtype of marginal zone lymphoma; FL, follicular lymphoma; HCL, hairy cell leukemia; LPL, lymphoplasmacytic lymphoma; MALT, mucosa‐associated lymphoid tissue; MCL, mantle cell lymphoma; SLL/CLL, small lymphocytic leukemia/small lymphocytic leukemia; SMZL, splenic marginal zone lymphoma. +, >90% positive; +/−,>50% positive; −/+,<50% positive; and −,<10% positive.

Expression of cyclin D1 is seen in more than 95% of cases. Cytogenetic analysis by Fluorescence in situ hybridization (FISH) will show the classic chromosome translocation, t(11;14) (q13;q32) in most cases.

*Cyclin D1 negative MCL*: This is a rare type of MCL identifiable by its lack of Cyclin D1 immunophenotypic expression or gene rearrangement. They are identical in morphology, immunophenotype (except Cyclin D1 expression) and clinical features to the classical MCL. Comprehensive gene expression profiling can confirm them as MCL, while molecularly, most demonstrate CCND2 (accounting to around 50% cases) or CCND3 gene rearrangements.^14^ They are positive for SOX11.^15^

Staging by good quality contrast enhanced imaging of chest, abdomen and pelvis, is recommended. Whole body PET‐CT, though not mandatory, is a common staging modality and has replaced conventional CT based staging in many centres. Staging is important not only to determine the extent of disease, but also to decide an appropriate treatment strategy. A lumbar puncture and CSF cytology is recommended in patients with blastoid morphology or neurological signs and symptoms. Patients with significant gastrointestinal symptoms must be evaluated with endoscopic examination (and biopsies) where appropriate to rule out involvement. MCL is commonly staged now using the Lugano classification for Non‐Hodgkin Lymphoma (Table 2).^16^

**TABLE 2:** Revised staging system for primary nodal lymphomas

Stage	Involvement^a^	Extranodal status^b^
I	One node or a group of adjacent nodes	Single extranodal lesions without nodal involvement
II	Two or more nodal groups on the same side of the diaphragm	Stage I or II by nodal extent with limited contiguous extranodal involvement
II (bulky)^c^	as above with “bulky” disease	Not applicable
III	Nodes on both sides of the diaphragm; nodes above the diaphragm with spleen involvement	Not applicable
IV	Additional noncontiguous extralymphatic involvement	Not applicable

*Note*: Lugano classification for non‐Hodgkin lymphoma used for staging of MCL.

a PET‐CT is used for avid lymphomas and CT for nonavid histologies to determine the extent of disease.

b Tonsils, Waldeyer's ring, and spleen are considered nodal tissue.

c Stage II bulky disease is treated as limited or advanced disease based on histology and number of prognostic factors.

## 5 PROGNOSIS

Several markers have been evaluated to predict the survival outcomes in MCL. Some of the currently recognized prognostic markers include performance status, CNS involvement at diagnosis, transformed MCL status, blastoid/pleomorphic morphology, Mantle cell International Prognostic index (MIPI) High risk group, Ki 67% >30%, Complex karyotype, Tp53 mutations or overexpression, MYC translocation or overexpression and unmutated IGHV status. Many novel markers like the CCND1, NOTCH 1 and 2 mutations, bruton tyrosine kinase (BTK), MAP3K14, CARD11, PCR for t(11;14), and so forth are being actively explored.^4^, ^17^ Among all known prognostic markers, blastoid morphology, TP53 mutation, complex karyotype and high Ki‐67 score are clearly associated with poor prognosis.^18^, ^19^, ^20^ The simplified MIPI, which is commonly used in practice, is shown in Table 3.^21^, ^22^

**TABLE 3:** Simplified mantle cell International Prognostic Index (MIPI)

Points	Age (years)	ECOG PS	LDH (UNL)	WBC (×10^9^/L)
0	<50	0–1	<0.67	<6.700
1	50–59	–	0.67–0.99	6.700–9.999
2	60–69	2–4	1.00–1.49	10.000–15.000
3	70	–	1.500	>15.000

Risk stratification based on MIPI score and their overall survival rates		
SCORE	Risk stratification	OS in months (5 year OS %)
0–3	Low risk	NR (60%)
4–5	Intermediate risk	51 months
6–11	High risk	29 months

*Note*: Simplified mantle cell International Prognostic Index (MIPI).

Abbreviations: ECOG PS, Eastern Cooperative Oncology Group performance status; LDH, lactate dehydrogenase; OS, overall survival; UNL, upper normal limit; WBC, white blood cells.

## 6 MANAGEMENT OF MANTLE CELL LYMPHOMA

MCL is an aggressive disease and is known to exhibit short durations of response, early relapse, shorter progression free survival (PFS) and overall survival (OS) compared to other NHLs.^23^ The goals of therapy in MCL are symptom reduction, disease control, improvement in quality of life, and cure when feasible. While choosing appropriate management strategy for patients with MCL, one needs to consider the following factors: assessment of physical fitness (performance status, or frailty in the elderly), comorbidities, disease stage, blastoid /non‐blastoid morphology, Ki‐67/MIPI score, access to therapy in various countries, cost‐coverage and availability of ongoing clinical trials.^24^

### 6.1 Initial management of mantle cell lymphoma

MCL patients can present with Indolent or aggressive disease based on clinical presentation and prognostic markers. (Figure 1) The management approach differs accordingly.

Indolent MCL patients having a good performance status, asymptomatic disease (no or minimal symptoms, low volume lymphadenopathy, lymphocytosis and splenomegaly), no high‐risk prognostic markers and/or low risk MIPI can be managed with “Watchful waiting” without a need for immediate systemic therapy. Such patients can be put under observation and carefully monitored for appearance of signs and symptoms of progressive disease. The MCL Biobank Observational Study by McCulloch et al, evaluated 315 MCL patients and showed that 67.3% of patients received upfront systemic therapy at baseline, 4.1% received localized radiation therapy and 27.6% of the patients were put on wait and watch strategy, 90 days beyond diagnosis. Of the 87 patients put on wait and watch strategy, 73.5% of them were under observation at 1 year, and 50.6% at end of 2 years. The study demonstrated the high prevalence of wait and watch strategy in clinical practice and reassured clinicians that half of them remained on observation beyond 2 years. However, the study also demonstrated the need for better predictive markers for indolent MCL.^25^

In the aggressive MCL, treatments depend on the extent of disease. In early‐stage disease (Stage1A and 2A), the evidence for management is scarce due to the small numbers of patients. Involved field radiotherapy (IFRT) can achieve up to 80% complete remission (CR) rates and long‐term remission, with potential cure in some patients.^26^, ^27^ In advanced stage disease, most patients need systemic chemoimmunotherapy. Indications for treatment include symptomatic disease (B symptoms, symptomatic organomegaly, organ dysfunction, GI symptoms including bleeding, and so forth), bulky disease, and bone marrow failure (significant cytopenia), among others.

### 6.2 First Line therapy for mantle cell lymphoma

Once a decision to treat the patient is made, patients who are treatment naïve are initially given induction therapy followed by maintenance therapy. There are two approaches in induction therapy, depending on the fitness of patients, based on current guidelines.^28^, ^29^ In the fit patients, an intensive therapy approach consists of high dose cytarabine containing induction regimens like Hyper‐CVAD + R/Mtx‐HA, R‐DHAP, etc.^30^, ^31^ This results in PFS and OS rates in the range of 73% and 61% at 46 months respectively.^32^ Once a response is achieved with induction therapy, the fit patients must be considered for consolidation therapy with high‐dose chemotherapy (HDT) like BEAM and autologous stem cell transplantation (ASCT). They receive maintenance therapy with Rituximab monotherapy for 3 years thereafter. In the patients who are not fit, a less aggressive approach with RCHOP or BR is preferred. Maintenance rituximab is administered without a post‐induction HDT‐ASCT consolidation in these patients. Outcomes in this setting are conservative and yield a PFS and OS rate in the range of 54.7 and 69.6 months, respectively.^33^ Maintenance therapies are not without controversy.^34^ Specifically, it is difficult to recommend an optimum duration or regimen for maintenance therapy. In the European MCL Network Study, MCL Elderly Study,^35^ treatment was continued until disease progression; however, many institutions limit the frequency to rituximab once in 3 months and the duration to 2 years.

Currently there are many studies that aim to improve upfront therapy in the young and fit patients by incorporation of targeted agents already known to have activity in relapsed setting. These agents include ibrutinib, lenalidomide, bortezomib and others. They are incorporated into the induction phase, maintenance phase, or both phases of treatment. A similar approach in the elderly has been tested in the Nordic MCL4 (LENA‐BERIT) study. Treatment naïve elderly patients (>65) or younger patients unfit for intensive therapy were treated with BR plus lenalidomide for six cycles followed by maintenance lenalidomide for a total of seven cycles. Despite a high response rate, this study noted significantly higher toxicity.^36^ A recent update from the European MCL Elderly trial, which evaluated R‐CHOP versus R‐FC in 560 newly diagnosed MCL patients, showed that, at a median follow‐up of 7.6 years, R‐CHOP showed superior benefits in terms of OS and median PFS. Rituximab maintenance following R‐CHOP had lesser toxicity than after R‐FC regimen.^37^ In the Phase2 WINDOW‐1 study, 131 patients were given ibrutinib‐rituximab (IR) induction (part‐A) until they achieved CR, for a maximum of 12 cycles. This was followed by a maximum of 4 cycles of Hyper‐CVAD + R/Mtx‐HA as consolidation therapy (part‐B). The study showed that frontline treatment with IR followed by a short course Hyper‐CVAD + R/Mtx‐HA is extremely potent and safe in patients aged ≤65 years with MCL^38^ Table 4 summarizes the important trials supporting use of therapies in aggressive and less aggressive settings. The commonly used aggressive and less aggressive therapy are listed in Table 5.

**TABLE 4:** Suggested treatment regimens for first line and supporting references

Treatment	Comparator	Sample size (*n*)	Median follow‐up	ORR	CR	PFS	OS	Safety/AE	Reference
Aggressive therapy									
Hyper CVAD + R		97	40 mo	97%	87%	64%	82%	Hematologic myelodysplasia/AML	32
		63	46 mo	83%	72%	73%	61%		39
Nordic regimen with Maxi‐CHOP		160	6 years	96%	54%	66%	70%	Neutropenic fever Infections Heart failure	40
RCHOP/RDHAP		60	67 mo	95%	57%	83mos	75%	Renal toxicity Neurologic toxicity	41
RDHAP		299	50.2 mo	89%	77%	83%	89%		42
Less aggressive therapy									
BR	R‐CHOP	274	45 mo	–	–	69.5 mo	–	Erythematous skin reactions	43
	R‐CHOP/R‐CVP	447	–	97%	31%	–	–	Vomiting Drug hypersensitivity	44
BR+ R(maintenance)	BR	120	54.2 mo	–	–	54.7 mo	69.6 mo		33
VR‐CAP	R‐CHOP	487	82 mo	–	–	–	90.7 mo	Infections Cardiogenic shock Acute renal failure Pulmonary carcinoma	45
R‐CHOP	FCR	560	37mo	–	34%	–	62%	Constipation Neuropathy Febrile neutropenia	37
Modified Hyper CVAD + R(maintenance)	–	22	37 mo	77%	64%	37 mo	NR		46
L + R	–	38	64 mo	–	–	80%	90%	Cytopenias infections	47
RBAC500	–	57	–	–	91%	–	–	Neutropenia, thrombocytopenia Fatigue nausea vomiting	48
Maintenance therapy									
Rituximab	R‐CHOP vs FCR	560	7.6 years	–	–	5.4 years	9.8 years	High incidence of death in remission Leukopenia infection	37

*Note*: Summary of important Trials supporting use of different regimens in management of treatment naïve MCL.

Abbreviations: BR, bendamustine + rituximab; Hyper CVAD, cyclophosphamide vincristine doxorubicin dexamethasone alternating with high dose methotrexate and cytarabine; L + R, lenalidomide + rituximab; mo, months; NORDIC Regimen, dose intensifying induction immunochemotherapy with rituximab + cyclophosphamide vincristine doxorubicin prednisone (maxi CHOP) alternating with rituximab + high dose cytarabine; R, rituximab; RBCA500, rituximab bendamustine cytarabine; RCHOP, rituximab + cyclophosphamide + doxorubicin + vincristine + prednisone; RDHAP, rituximab + dexamethasone + cytarabine + cisplatin; VR‐CAP, bortezomib + rituximab + cyclophosphamide + doxorubicin + prednisone.

**TABLE 5:** Guidelines for management of mantle cell lymphoma

			ESMO guidelines (2017)	BSH guidelines (2018)			NCCN guidelines (2021)	
MCL patients	First Line	Young fit	Dose intensified chemoimmunotherapy RCHOP HD‐AraC Followed by ASCT Rituximab Maintenance	Suitable for ASCT	High dose cytarabine + rituximab Followed by ASCT Rituximab maintenance		Candidate for HDT/ASCR ‐ Aggressive therapy	**Preferred regimens** RDHA Alternating R‐CHOP/RDHAP NORDIC regimen Hyper CVAD + R ^a^ R + bendamustine > R + cytarabine Other recommended regimens BR **Consolidation after aggressive therap**y HDT followed by ASCT **Maintenance After HDT/ASCR** Rituximab every 8 weeks × 3 years ^a^ *Pre‐treatment with ibrutinib‐rituximab to reduce number of Hyper CVAD cycles (Window‐1 study)*
		Elderly frail	Conventional chemoimmunotherapy R‐CHOP VR‐CAP BR R‐BAC Followed by Rituximab maintenance	Not Suitable for ASCT	Fit for conventional immunotherapy	R‐CHOP BR VR‐CAP Followed by Rituximab maintenance	Not a candidate for HDT/ASCR ‐ Less Aggressive therapy	**Preferred regimens** BR VR‐CAP RCHOP L + R **Other recommended regimens** Modified R‐Hyper CVAD in >65 years patients RBAC500 **Maintenance therapy:** For RCHOP: Rituximab every 8 weeks until PD or intolerance For Hyper CVAD + R: rituximab every 8 weeks × 2–5 years
		Compromised	Best supportive care R‐chlorambucil BR (dose‐reduced) R‐CVP		Unfit for conventional immunotherapy	Low intensity immunochemotherapy		
	Relapse/ Refractory	Young fit	Immunochemotherapy R‐BAC BR Targeted approaches Followed by AlloSCT	Suitable for ASCT	BTK inhibitor R‐BAC R‐CHOP BRLater: Consider AlloSCT		Partial response with intention to proceed to transplant	Preferred regimens B ± R Bortezomib ± R L ± R RCHOP or VR‐CAP
		Elderly frail	Immunochemotherapy R‐BAC BR targeted approaches Followed by Rituximab maintenance or Radioimmunotherapy	Not Suitable for ASCT	BTK inhibitor R‐BAC R‐CHOP BR		Short response duration to prior CIT (< expected median PFS)	**Preferred regimens** BTK inhibitors Acalabrutinib Ibrutinib ± R Zanubrutinib L ± R **Other recommended regimens** Ibrutinib, L, R Venetoclax + ibrutinib
		Compromised	Immunochemotherapy R‐BAC BR targeted approaches				Extended response duration to prior CIT (> expected median PFS)	**Preferred regimens** B ± R Bortezomib ± R BTK inhibitors Acalabrutinib Ibrutinib ± R Zanubrutinib L ± R **Other recommended regimens** Venetoclax ± R B, Bortezomib and R PEPC RCHOP or VR‐CAP DHAP or GemOx, ± R
	Higher Relapse		Targeted approaches: (preferable in combination with chemotherapy) ibrutinib lenalidomide Temsirolimus, Bortezomib Alternatively: repeat previous therapy (long remissions)	Higher Relapse	Alternative immunochemotherapy, BTK inhibitor or other targeted therapy		Second Line Consolidation	AlloSCT

*Note*: Recommendations from ESMO, BSH and NCCN guidelines for management of MCL. Refer Tables 4 and 6 for abbreviations.

*Role of CNS prophylaxis*: The ability of CNS‐penetrating therapies to reduce the incidence of CNS relapse is unclear, however empirical use of CNS‐penetrating chemotherapy may be beneficial in patients with high Ki67 and/or blastoid morphology. CNS relapse rates of 1.6%–25.4% are reported mainly in patients with high Ki67 and blastoid histology.^49^ CNS relapse tends to occur early within a median duration of 15–20 months and in them survival is poor (3–8 months). Ninety percent of CNS relapses are leptomeningeal disease confirmed by flow cytometry, and parenchymal disease is rare.^50^

### 6.3 Stem cell transplantation in mantle cell lymphoma

Autologous stem cell transplantation (ASCT) and the Allogenic stem cell transplantation (AlloSCT) have been used in the treatment of mantle cell lymphoma.^51^

#### 6.3.1 Autologous stem cell transplantation

ASCT is generally advised in the fit MCL patients to consolidate a response to induction therapy. Superior outcomes are noticed when patients are treated with ASCT while in first CR or in a minimal disease state and have not received multiple prior chemotherapy regimens.^52^ The HDT regimens routinely used are BEAM or TBI based. Patients, who do not achieve CR despite multiple chemotherapy regimens, are not ideal candidates for ASCT. As the treatment landscape of MCL is quickly evolving, it would be appropriate to re‐visit the role of ASCT in real‐world practice soon.^53^ There is an active interest in radio immunotherapy in some groups.^54^ Whether ASCT can be safely omitted from intensive first‐line therapy that incorporates a Bruton's tyrosine kinase (BTK) inhibitor, is being tested in the European MCL Network TRIANGLE trial (NCT02858258): ASCT after a rituximab/ibrutinib/Ara‐c containing induction in mantle cell lymphoma.^55^

#### 6.3.2 Allogenic stem cell transplant

AlloSCT is an attractive therapeutic modality in MCL due to a potential benefit from a graft versus lymphoma effect, and avoidance of marrow contamination by the tumor as possible in ASCT.^56^, ^57^ Standard AlloSCT is yet not considered in the upfront treatment setting. This is due to the improved results from ASCT, advanced age at presentation for most patients, toxicity and higher non‐relapse mortality from AlloSCT, and an overall paucity of data. Currently, AlloSCT is considered only in the setting of relapsed refractory MCL and younger fit patients. Reduced intensity conditioning regimens are the preferred preparative treatment in such a scenario.

### 6.4 Therapy for relapsed and refractory mantle cell lymphoma

Despite excellent responses following primary therapy (60%–97%), it is exceptional to experience long‐term relapse free survival in mantle cell lymphoma. The duration of responses hinges considerably on the aggressiveness of the disease at presentation and type of initial treatment used. Most patients relapse and progress to refractory disease.^58^ Most second line therapies for relapsed and refractory (R/R) MCL do not offer cure, with the potential exception of allogenic stem cell transplantation. Therefore, almost always, the goal of therapy remains to palliate symptoms, gain control and prolong remission rates and progression free survival. There have been a few agents, used as monotherapy or in combination strategies, explored with limited efficacy and backed by insufficient data.^59^ The past decade witnessed the use of non‐chemotherapy options to perturb pathways or the microenvironment. It saw the FDA approval of five such agents in relapsed refractory MCL including bortezomib, lenalidomide, ibrutinib, acalabrutinib and zanubrutinib.

Prior to the advent of targeted approaches to treat R/R MCL, salvage chemotherapy was the standard for treating relapsed mantle cell lymphoma. The initial use of gemcitabine, in combination with platinum (cisplatin and oxaliplatin) resulted in PFS rates ranging from 85 to 22 months.^60^, ^61^ The later use of bendamustine with rituximab also yielded similar PFS rates.^61^, ^62^ The improvement in survival came with the exploration of targeted therapy with or without chemotherapy/ immunotherapy. The retrospective MANTLE‐FIRST study recently reported on the outcomes of a large cohort of patients with first relapsed‐refractory MCL after upfront intensive high‐dose cytarabine based therapy. Bendamustine based regimens (50%) and Ibrutinib (19%) were the common first salvage treatments received. Though overall outcomes were superior in the patients who received salvage rituximab‐bendamustine‐cytarabine (R‐BAC) and ibrutinib with a median PFS ranging around 24 months, there was a significant benefit with Ibrutinib in patients who had early relapse or progression (defined as <24 months from diagnosis).^63^

The past decade saw the emergence of non‐chemotherapy approaches including bortezomib, temsirolimus, rituximab, lenalidomide, ibrutinib, acalabrutinib and zanubrutinib into the therapeutic armamentarium. More recently, BCL2 inhibitor‐venetoclax and CDK inhibitor‐palbociclib are being explored. The currently approved drugs/regimens for R/R MCL include Bendamustine, R‐CHOP, temsirolimus, rituximab, bortezomib, lenalidomide, ibrutinib, acalabrutinib and zanubrutinib. Regimen and evidence supporting their use in R/R MCL are discussed in Table 6.

**TABLE 6:** Suggested treatment regimens for second line and supporting references

Treatment	Comparator	Sample size (*n*)	Median follow‐up	ORR	CR	PFS	OS	Safety/AE	References
BR	–	67	92%	–	–	23 mo	–	Myelosuppression	[61]
BR + bortezomib	–	29	–	83%	51.7%	47%	–	Nausea Neuropathy Fatigue Constipation Fever	[64]
Bortezomib + R (PINNACLE study)	–	155	26.4 mo	–	–	6.7 mo	23.5 mo	Peripheral neuropathy Lymphopenia	[65]
CHOP versus Bortezomib Plus CHOP	CHOP	46	34	48.8% versus 82.6%	21.7% versus 34.8%	8.1% versus 16.5 mo	11.8 versus 35.6 mo	Sensory neuropathy similar in both arms	[66]
Lenalidomide (L)	–	57	–	35%	12%	8.8 mo	NR	Neutropenia Thrombocytopenia anemia	[67]
L + R	–	52	–	57%	36%	11.1 mo	24.3 mo	Hematologic toxicities Febrile neutropenia	[68]
PEP‐C	–	22	–	82%	46%	–	–	Myelosuppression Nausea vomiting	[69]
Ibrutinib	Temsirolimus	139	38.7 mo	77%	23%	26.2 mo	30.3 mo	Hematological toxicities Diarrhea Fatigue Cough URTI	[70]
Ibr + R	–	50	16.5 mo	88%	44%	69%	83%	Atrial fibrillation Diarrhea Neutropenia infections	[71]
Acalabrutinib	–	124	15.2 mo	81%	40%	67% (12 mo)	87% (12 mo)	Headache Diarrhea Fatigue	[72]
Zanubrutinib	–	86	13.9 mo	84.7%	76.5%	16.7 mo	–	Neutropenia URTI Leukopenia Thrombocytopenia Hypokalemia Diarrhea Hypertension	[73]
Orelabrutinib	–	106	–	82.5%	24.7%	NR	NR	Thrombocytopenia Neutropenia Respiratory tract infections Rash	[74]
Pirtobrutinib	–	323	6 mo	52%	NR	NR	NR	Fatigue, diarrhea, contusion Neutropenia	[75]

*Note*: Summary of important trials supporting use of different regimens in management of relapse and refractory mantle cell lymphoma.

Abbreviations: BR, bendamustine + rituximab; Ibr, ibrutinib; L, lenalidomide; mo, months; PEP‐C, prednisolone etoposide procarbazine cyclophosphamide; R, rituximab.

The ability of bortezomib to induce tumor cell apoptosis in lymphomas primarily through NFKB inhibition and caspase independent mechanisms led to its exploration in R/R MCL.^76^ The phase II PINNACLE trial used bortezomib in combination with rituximab showing an overall response rate of 32% with 8% CR/uCR (unconfirmed CR) with median time to progression (TTP) of 6.7 months and a median OS of 23.5 months in an updated analysis.^65^ Further combinations, with chemotherapeutic agents, improved PFS although at the expense of some increased toxicity.^64^ Prior treatments with neurotoxic chemotherapeutic agents increased the incidence of neuropathy.^77^

Lenalidomide as an immunomodulatory agent demonstrated notable activity in mantle cell lymphoma through it antineoplastic and antiproliferative effects. The NHL 003 study, which also included R/R MCL, showed a 35% ORR with 12% CR/ uCR with a median duration of response (DoR) of 16.3 months, and median PFS of 8.8 months.^67^ The definitive role for lenalidomide in R/R MCL was explored in the phase II MCL001, EMERGE study. The ORR was 38% with CR/ uCR being 8%, and a median DoR, PFS and OS at 16.6, 4 and 20.9 months, respectively. An exploratory analysis in the study demonstrated activity for lenalidomide irrespective of the Ki67 status although OS was significantly lower for groups with <30% Ki67 versus those with higher (9.7 vs. 28.4 months respectively).^78^ The addition of Rituximab to lenalidomide improved ORR to 53% with a CR of 31%, and a median OS of 14 months with duration of response lasting 18 months.^68^

The constitutive activation of B cell receptor signaling (BCR) is integral to survival and proliferation of malignant B cells. Inhibitors of B cell receptors associated kinase interfere with BCR signaling reducing tumor burden. Ibrutinib, a first in class BTK inhibitor reduced tumor burden in 7 out of 9 patients with R/R MCL in a phase I study.^79^ The follow up phase II study yielded unprecedented response rates of 68% (CR 21% and PR 47%) with median response lasting 17.5 months, median PFS of 13.7 months and updated OS of 22.5 months.^80^ The randomized Phase III study showed better ORR and longer PFS with better toxicity profile over comparator Temsirolimus.^81^ The pooled analysis of 370 patients treated on Ibrutinib in clinical trials showed a third of patients continuing on therapy for 2 years or more with about 10% still continuing on Ibrutinib at 4 years.^82^ The greatest impact was in the setting of first relapse with a median PFS of 33.5 months and DoR of 34.4 months.^83^ The combination therapies with Ibrutinib have generally not shown added benefits over monotherapy, this is however subject to debate. A phase II study of ibrutinib and rituximab (IR) combination did not improve outcomes in the initial report, though there was a higher ORR in patients with lower Ki‐67 (<50% vs. >50%).^71^ A recent update from a longer follow‐up of the same IR cohort reports durable remissions with 58% achieving CR, mPFS of 43 months, 3 year PFS of 54% and 3 year OS of 69%, at a median follow up of 47 months. The patients with lower Ki67 had a 3 year OS of 67% compared to 27% for Ki67‐high group.^84^ The PHILEMON study explored the combination of Ibrutinib with Lenalidomide and Rituximab and reported an ORR of 76% and CR of 56% at a median follow‐up of 17.8 months.^85^ The toxicity profile was however high with frequent neutropenia, infections and cutaneous reactions.

The next generation BTK inhibitor, acalabrutinib, a more selective BTK inhibitor was designed to mitigate the ‘off‐target action’ induced adverse effect profile encountered with ibrutinib. The phase II study involving 124 patients with R/R MCL achieved an ORR of 81% with CR rates of 40%. The PFS at 12 months was 72% with OS of 87%. There were lower side effects (6%) encountered with progressive disease (31%) being the primary reason for discontinuation.^71^ Similar results were seen with another selective BTK inhibitor, zanubrutinib, which received accelerated FDA approval based on the impressive results from phase II and phase I/II clinical trials yielding ORR of 84% (CR 59% and PR 24%) in relapsed MCL previously treated with one line therapy.^72^, ^86^

Venetoclax, a BCL2 inhibitor has been used effectively in a range of hematological malignancies with impressive results particularly in chronic lymphatic leukemia when used in combination with ibrutinib. As a single agent in MCL the ORR was 75% (CR 21%) and a PFS of 14 months.^87^ Two other venetoclax monotherapy studies report ORR in the range of 50%–60%, with nearly 21% achieving CR at follow‐up periods of up to 17 months. The mPFS and OS were in the range of 2.6–8 months and 4.3–13.5 months, respectively.^88^, ^89^ Given its synergy with ibrutinib, the AIM study explored this combination with a lead‐on period for ibrutinib followed by addition of venetoclax. The CR at 16 weeks of therapy was 42% with a measurable residual clearance of 67% in the bone marrow assessed by flowcytometry. In an updated analysis with a follow up of 37.5 weeks the median DoR and TTP were not reached. The median PFS and OS was 29 and 32 months respectively.^90^

Given the invariable resistance encountered for Ibrutinib in MCL, palbociclib, an oral CDK4/6 inhibitor capable of overcoming primary ibrutinib resistance was explored in combination with ibrutinib.^91^ The phase I study demonstrated an ORR of 67% with 37% and a 2‐year PFS of 59%.^92^ The study gave credence for this rationale behind the combination, and further studies are underway.

The future of treatment for mantle cell lymphoma particularly in the R/R setting is evolving. The use of small molecules for treating relapsed MCL is now established, with diminishing role for conventional chemotherapy. The emerging role for CAR‐T cell and other immunotherapy‐based approaches (discussed separately) will redefine MCL treatments. The key lies in defining subsets for optimized outcomes in subgroup of patients.

## 7 GUIDELINE RECOMMENDATIONS FOR MANTLE CELL LYMPHOMA

Current recommendations from the European Society for Medical Oncology (ESMO),^28^ British Society for Hematology (BSH)^24^ and National Comprehensive cancer Network NCCN^29^ are summarized in Table 5.

## 8 FUTURE TRENDS IN MANAGEMENT OF MANTLE CELL LYMPHOMA

### 8.1 Newer Bruton's tyrosine kinase inhibitors

Ibrutinib use is associated with certain toxicities in the long term like higher rates of cardiac arrhythmias, hypertension, and risk of bleeding. These and emergent resistance lead to discontinuation of the therapy in many patients.^93^ To overcome these, BTK inhibitors with higher specificity, the second‐generation BTK inhibitors like acalabrutinib, zanubrutinib, orelabrutinib, tirabrutinib and pirtobrutinib (formerly LOXO‐305) have emerged.^94^, ^95^, ^96^, ^97^ As discussed above, the second‐generation BTK inhibitors have much better selectivity, reducing the off‐target effects of the drugs.^98^ When considering the currently available BTK inhibitors, emerging data suggest that acalabrutinib and tirabrutinib may have higher kinase selectivity than other available BTKi's.^99^

### 8.2 Chimeric antigen receptor T‐cell therapy in mantle cell lymphoma

Chimeric antigen receptor (CAR) T‐cell therapy is one of the major advances in synthetic biology and successful examples of personalized cancer therapy to be made commercially available in the recent past. This therapy has shown success in hematologic cancers with reduction in remission rates of up to 80%, particularly for ALL and DLBCL, and received FDA approval.^100^, ^101^ In July 2020, brexucabtagene autoleucel (KTE‐X19), a CD19‐directed genetically modified autologous T‐cell immunotherapy was granted accelerated approval by US‐FDA for the treatment of adult patients with relapsed or refractory mantle cell lymphoma.

The approval followed the impressive results from the phase II multicentric ZUMA‐2 trial evaluating KTE‐X19 in 74 patients with relapsed/refractory mantle cell lymphoma previously treated with a BTK inhibitor. Of the 74 patients enrolled, 68 received KTE‐X19 with a median follow‐up of 12.3 months (range = 7 to 32.3 months). The Objective response rate was 93% (95% CI 84–98), and the complete response 67% (95% CI 53–78). 57% of patients were in remission at 12 months. The estimated PFS was 61% and OS was 83% at 12 months. Cytopenia (94%), infection (32%), neurologic events (31%) and cytokine release syndrome (15%) were the common >grade 3 adverse events encountered during the trial (all resolved successfully). There were two deaths due to infections during the trial. The study showed that KTE‐X19 is an effective and viable option for patients with R/R MCL with a manageable safety profile.^102^

In line with CAR‐T Cell therapies, newer modalities of adoptive cellular therapies like next generation CAR strategies including dual CAR and multi‐CAR T‐cell therapies, natural‐killer (NK) cell‐based therapies, dendritic cell therapies, and so forth are in early phases of clinical testing across the world.

### 8.3 Novel combination therapies in mantle cell lymphoma

Combinations of novel therapies with conventional as well as monoclonal antibody‐based therapies hold the potential of deepening response in MCL patients and improve efficacy. Many such combination therapies have been tried in clinical trials. They are summarized in Table 7.

**TABLE 7:** Summary of important Trials supporting combination therapies in MCL

Treatment	Phase and Comparator	Sample size (n)	Median Follow‐up	ORR	CR	PFS	OS	Safety	Reference
I + R	Single arm Phase 2	50	47 mo	88	58%	43 mo	NR	Fatigue Diarrhea Nausea Arthralgias myalgias	[84]
I + L + R	Single arm Phase 2	50	17.8 mo	76%	56%	16 mo	22 mo	Neutropenia Infections Cutaneous toxicities	[85]
I + Ven	Single arm with Historical controls Phase 2	24	15.9 mo	71%	71%	NR	NR	Diarrhea Fatigue Nausea/Vomiting	[103]
I + Palb	Single arm Phase 1	27	25.6	67%	37%	59.4%	NR	Neutropenia Thrombocytopenia Hypertension Febrile neutropenia Lung infection	[92]
I + Obi +Ven	Single arm Phase 1	15	–	100%	46.6%	NR	NR	Hepatobiliary disorders Rash Hematological toxicities	[104]
I + BR	Single arm Phase 1/1b	17	–	94%	76%	NR	NR	Lymphopenia Neutropenia Thrombocytopenia Rash	[105]
Acalabrutinib + B + R (Treatment naïve)	Single arm Phase 1b	18	17.6mo	94%	72%	NR	NR	Neutropenia Pneumonia Nausea Fatigue Vomiting	[106]
Acalabrutinib + B + R (relapse/Refractory)	Single arm Phase 1b	20	14.2 mo	80%	65%	16.6 mo	NR	Neutropenia Pneumonia Nausea Fatigue Vomiting	[106]

*Note*: Summary of important trials supporting combination therapies in MCL.

Abbreviations: BR, bendamustine rituximab; I, ibrutinib; L, lenalidomide; mon, months; NR, not reported; Obi, obinutuzumab; Palb, palbociclib; R, rituximab; Ven, venetoclax.

## 9 MANTLE CELL LYMPHOMA IN A MIDDLE‐INCOME SETTING, INDIA

**FIGURE 2:**
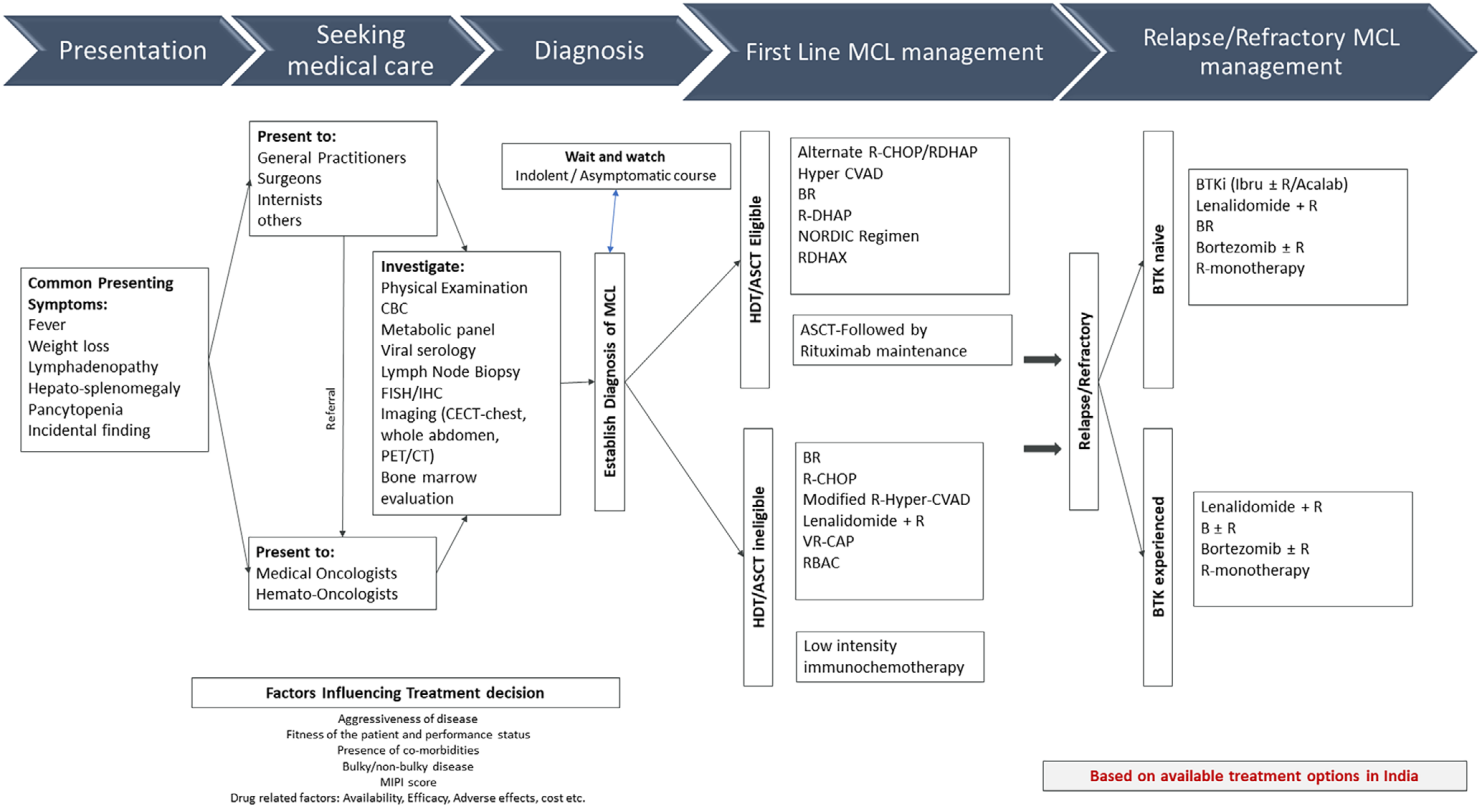
Schematic algorithm of management of MCL with currently available treatment options in India. BR, bendamustine + rituximab; CBC, complete blood counts; CECT, contrast enhanced computed tomography; CT, computed tomography; FISH, Fluorescent In‐situ Hybridization; Hyper CVAD, cyclophosphamide vincristine doxorubicin dexamethasone alternating with high dose methotrexate and cytarabine; IHC, immuno‐histo‐chemistry; L + R, lenalidomide + rituximab; NORDIC Regimen, dose intensifying induction immunochemotherapy with rituximab + cyclophosphamide vincristine doxorubicin prednisone(maxi CHOP) alternating with rituximab + high dose cytarabine; PET, positron emission tomography; R, rituximab; RBAC, rituximab bendamustine cytarabine; RCHOP, rituximab + cyclophosphamide + doxorubicin + vincristine + prednisone; RDHAP, rituximab + dexamethasone + cytarabine + cisplatin; VR‐CAP, bortezomib + rituximab + cyclophosphamide + doxorubicin + prednisone

A review of literature on MCL from India is constrained by the paucity of data. The rarity of the disease poses challenges in its reporting. One retrospective epidemiological study looking at the distribution of different non‐Hodgkin lymphomas in India showed that MCL accounted for 1.59%–3.4% of NHL in India.^107^ A registry study by Naresh et al. which investigated lymphoid malignancies from three different regions in India reported 562 lymphoid malignancies of which 386 (68.6%) were NHL. They noted differences in relative frequencies of MCL in the 3 regions where MCL accounted for 0% cases of NHLs in rural Barshi, 4.6% (95%CI: 0.2%–9.0%) in Pondicherry and 1.9% (95%CI: 0.1–3.7%) in Jaipur.^108^ A retrospective case series comprising of 13 MCL cases from a tertiary care center in Southern India over a period of five and half years showed that MCL accounted for 4% of all NHLs, with a male preponderance (2.25:1) and a median age of presentation of 57 years.^109^ Another retrospective study of 93 MCL cases, over a period of 4 years, showed that MCL accounted for 2.1% of all NHLs with a median age of presentation of 57 years and a male preponderance (3.8:1). The study concluded that although the incidence of MCL is low compared to western population, incidence and frequency of the usual morphological variants and subtypes of nodal and extra nodal MCL is similar.^110^ A prospective study to determine the prevalence of t^11^, ^14^ in healthy volunteers by nested polymerase chain reaction (PCR) on peripheral blood samples reported a prevalence of 0.48% among 210 samples.^111^ A similar study from the west reported a prevalence of 1%.^112^ Data on treatment outcomes in the Indian setting is limited to case‐reports, and a few case‐series, which have reported on mantle‐cell lymphoma as a part of a cohort of NHL. The largest retrospective series of 51 patients, from a single centre, reported an earlier age at presentation (median = 57 years), frequent use of Cyclophosphamide, doxorubicin, vincristine, prednisolone (CHOP) based chemotherapy with or without Rituximab, 3‐year overall survival (OS) rate of 54%, 2‐year progression free survival (PFS) of 27%, and improved outcomes with the addition of rituximab to chemotherapy.^113^ The smaller case series document the use of ibrutinib,^114^ and lenalidomide,^115^ in relapsed or refractory MCL. On perusal of the clinical trials registries of India (www.ctri.nic.in) and the national institutes of health (www.clinicaltrials.gov), we found only five clinical studies in various phases. There were five pharma‐sponsored, and no investigator‐initiated studies. These were three novel therapy based clinical trials evaluating P‐276‐00 (a cyclin‐dependent kinase inhibitor), Ibrutinib and Acalabrutinib in Indian population. (www.ctri.nic.in). Two large ongoing consortium‐based registry programs under the aegis of Oncocollect® database, and Hematology cancer consortium® are expected to report soon on lymphoma outcomes from India. Middle‐income countries and their emerging economies will drive an aspirational populace to seek incorporation of novel therapies in the treatment of hard‐to‐treat cancers like MCL. Cost–benefit analyses will be key to their successful use. A schematic algorithm of management of MCL based on currently available treatment options in India is shown in Figure 2.

## 10 CONCLUSION AND FUTURE CHALLENGES

Management of MCL is bereft with challenges due to its resistant and relapsing pattern. Despite improvements in remission durations, the disease is currently incurable with standard therapy with a median survival of 3–5 years. As a biological entity, MCL is increasingly being considered as a heterogenous disease and this needs further understanding. The asymptomatic patients with (non‐nodal) indolent disease can be managed by close surveillance. In the aggressive variety, there is no single standard of care approach. Treatment decisions and iterations are based on disease biology and fitness of patients. Many studies today are aiming to improve upfront therapy by incorporation of targeted agents already known to have activity in relapsed setting and are proving to have better efficacy and tolerability than conventional chemoimmunotherapy. The future of treatment for mantle cell lymphoma particularly in the relapsed setting is an area of continuous movement. The use of small molecule inhibitors for treating relapsed MCL is increasingly established, with a diminishing role for chemotherapy. Combinations of novel therapies in MCL show promise and hope for better outcomes in MCL patients. Immunotherapy approaches like CAR‐T cell therapy, though approved now, need long‐term studies. Middle and lower‐middle income countries like India have unique challenges. As an increasingly aspirational society and intuitively, there is an urgent need for a systematic approach to determine the disease burden and current outcomes in MCL and other lymphomas. This can be achieved through ongoing registry efforts and prospective studies incorporating novel therapeutics, with a focus on cost–benefit analyses and patient reported outcomes.

## CONFLICT OF INTEREST

Vivek S. Radhakrishnan reports advisory fees (institutional) and non‐financial Institutional support from PFIZER, Institutional grants and non‐financial support from INTAS Pharmaceuticals, Institutional grants from NATCO Pharmaceuticals, Institutional grants from ROCHE, Institutional grants from BMS, Institutional grants and non‐financial support from CIPLA Pharmaceuticals, Institutional grants from EMCURE, personal fees (institutional) from ASTRA ZENECA, non‐financial institutional support from Dr. REDDY's Laboratories, outside the submitted work. Prashanth S.P. is an employee of AstraZeneca Pharma India Limited. Other authors do not report any significant conflicts of interest with regards to the submitted work.

## ETHICAL STATEMENT

Not applicable.

## AUTHOR CONTRIBUTIONS

**Vivek S Radhakrishnan:** Conceptualization (lead); data curation (equal); formal analysis (lead); methodology (lead); project administration (lead); resources (lead); software (equal); supervision (lead); writing – original draft (lead); writing – review and editing (lead). **Padmaja Lokireddy:** Conceptualization (supporting); data curation (supporting); formal analysis (equal); methodology (supporting); resources (supporting); supervision (supporting); writing – original draft (equal); writing – review and editing (equal). **Mayur Parihar:** Conceptualization (supporting); data curation (supporting); formal analysis (equal); methodology (supporting); project administration (supporting); resources (supporting); supervision (supporting); validation (equal); writing – original draft (equal); writing – review and editing (equal). **Prashanth S P:** Conceptualization (supporting); data curation (equal); formal analysis (equal); funding acquisition (equal); methodology (supporting); project administration (supporting); resources (equal); software (equal); supervision (supporting); validation (equal); writing – original draft (equal); writing – review and editing (equal). **Hari Menon:** Conceptualization (supporting); data curation (supporting); formal analysis (equal); methodology (equal); project administration (supporting); resources (equal); supervision (equal); validation (equal); writing – original draft (equal); writing – review and editing (equal).

## Data Availability

Data sharing is not applicable to this article as no new data were created or analyzed in this study.
